# Coagulation of the Blood in Arteries for the Cure of Aneurisms

**Published:** 1853-04

**Authors:** 

**Affiliations:** Lyons


### SURGERY.

*Coagulation of the Blood in Arteries for the Cure of Aneurisms.*—By Dr. PRAVAS of Lyons. The plan which Dr. Pravas proposes consists in coagulating the blood in the arterial vessels by the injection of a few drops of a concentrated solution of perchloride of iron. This is done with either a gold or platinum trocar, very finely pointed, introduced obliquely through the walls of the vessel with a rotatory sort of motion. The instrument being furnished with a syringe, and the piston worked by a screw, so that the injection would not be thrown in jerks, and the quantity of liquid used measured accurately. Besides, the circulation in the vessel must be temporarily stopped, and some other precautions taken, which will be mentioned directly, after an account of the experiments performed.

1st. The carotid of a sheep having been exposed, the current of blood was interrupted by the finger and thumb, at two points distant one inch and a half or two inches; about a spoonful of blood was thus enclosed.

A puncture was made very obliquely in the walls of the vessel, and three or four drops of the perchloride of iron injected. For this purpose two turns of the screw were made, each turn ejecting about two drops of the fluid. Immediately after the injection of the salt, an increase in the density of the blood could be felt, and a clot forming rapidly. In four minutes the vessel could be left to itself, leaving off all compression; in fact, the clot did not change its position, and it could be felt in the same place for eight days subsequently.

2nd. A similar experiment performed on a horse gave the same result. The portion of the artery in which the circulation had been suspended was about three inches long, and could contain five teaspoonfuls of blood. Eight or ten drops of the solution were injected, Dr. Pravas having observed that it took nearly two drops of the solution to coagulate a teaspoonful of blood. In four minutes in the horse, as well as in the sheep, the clot was fully formed; it was firm and resisting, and was not displaced by the impulse of the blood for a quarter of an hour, at the end of which time that portion of the vessel subjected to the experiment was cut out and split; the inner surface was rough, and showed granulations and longitudinal striæ along the whole length occupied by the clot.

3rd. In another horse, a similar experiment was performed, with exactly similar immediate results, except that this animal was preserved for eight days, leaving the vessel exposed, so as to watch the progressive changes. It was observed that the induration of the carotid extended above and below the formative clot. At the end of eight days the horse was killed, and on examining the interior of the artery, three distinct clots were seen, which caused its obliteration for about eight inches and a half in extent. The middle clot corresponded to where the injection was introduced, and had a darker-colored and granular appearance, about an inch and a quarter long.

*Resumé:* After the injection of the perchloride of iron, four minutes and a half were sufficient to cause a clot in the carotids of these animals so consistent and adherent as not to be displaced by the impulse of the column of blood from the heart.

The above experiments have been witnessed by M. Lallemand, and M. Le Coq, directeur de l'Ecole de Lyon. Dr. Pravas is still pursuing his researches, and has made these first results public, so as to draw the attention of experimentalists and practitioners to this plan of obliterating arteries. So far, his observations have been purely experimental, and instituted in such a way as to prove directly the mode of action of the coagulating agent he employs. As to its application for the cure of aneurism in the human subject, the proceeding ought to be thus modified: it is into the aneurismal sac that the solution ought to be injected, having previously arrested the circulation below the sac—that is, between it and the capillaries. The quantity injected should be in proportion to the size of the sac, and the compression below should last four or five minutes. This, in Dr. Pravas's opinion will be sufficient to form an extensive clot, capable of plugging the artery and producing the same effect as ligature.—*Dublin Med. Press, from Presse Méd. Belge.*

*On the Manufactnre of Sponge Tents.* By Dr. Digby.—[The following directions are given :] "A piece of tolerably fine sponge, previously well dried, should be soaked in Mistura Acaciæ, and rolled up into a cylindrical form, somewhat in the shape of a small segar, tapering to a point at one end. The other, or thick end, must be rolled round a middling-sized awl, partly for the purpose of a leaving a central perforation into which the end of the instrument which carries it is to be inserted, and partly to fix it, while a piece of stout cord is wound tightly and closely around it from the thick end up to the point. By this means, the sponge is powerfully compressed into the cylindrical form above mentioned, and if well-dried, becomes as hard as a piece of wood, and retains its compressed state perfectly when the cord is removed. Any little projections or roughnesses may be trimmed off with a sharp knife; and, lastly, the tent is to be dipped several times in melted tallow, rendered harder by the admixture of a little white wax, until it has become thickly coated. A piece of string or tape is fastened to the lower or thicker end, to assist in removing it from the os uteri when expanded. The heat of the part soon melts the unctuous covering, and thus enables the tent to slide up in its own grease as it gradually melts, when otherwise it might have been difficult to introduce it. The secretions of the part slowly pervade the sponge, and dissolve the hardened gum with which it has been soaked, and the sponge gradually expands as it returns to its full size.

"Twelve hours is usually a sufficient period to affect this in; and the degree of dilatation produced will guide us to the introduction of a larger tent on the removal of the first."—*Med. Times.*

